# Acknowledgment to the Reviewers of *Journal of Functional Morphology and Kinesiology* in 2022

**DOI:** 10.3390/jfmk8010016

**Published:** 2023-01-28

**Authors:** Journal of Functional Morphology and Kinesiology Editorial Office

**Affiliations:** MDPI AG, St. Alban-Anlage 66, 4052 Basel, Switzerland

High-quality academic publishing is built on rigorous peer review. *JFMK* was able to uphold its high standards for published papers due to the outstanding efforts of our reviewers. Thanks to the efforts of our reviewers in 2022, the median time to first decision was 19 days and the median time to publication was 37 days. Regardless of whether the articles they examined were ultimately published, the editors would like to express their appreciation and thank the following reviewers for the time and dedication that they have shown *JFMK*:
Adamec, JiriLum, DannyAl-Dujaili, EmadMalça, CândidaAmbrosio, LucaMalliaropoylos, NikosAmbrozy, TadeuszManonelles, PedroAneta, TeległówMarchesini, MaurizioAntičević, DarkoMarotta, NicolaAyán Pérez, Cárlos L.Martin, EricBadau, DanaMartin, JoelBenítez-Martínez, Josep CarlesMartínez-González-Moro, IgnacioBentley, DavidMartinez-Jimenez, EvaBertrandt, JerzyMartínez-Rodriguez, AlejandroBESSON, PierreMascia, GiuseppeBirant, DeryaMassart, AlainBrancaccio, MariaritaMayda, HakanBrown, FreddyMazaheri, RezaBrumitt, JasonMcCurdy, KevinBuoite Stella, AlexMenegatti, EricaBurgos-Robles, AnthonyMennitti, CristinaCaby, IsabelleMinciacchi, DiegoCaldo-Silva, AdrianaMitschke, ChristianCalleja-Gonzalez, JulioModric, ToniCaravelli, SilvioMonteiro, António MiguelCarballeira, EduardoMontero Seoane, AntonioCassirame, JohanMorais, Jorge E.Casuso-Pérez, RafaelMorasiewicz, PiotrCavaggioni, LucaMotamed, CyrusCè, EmilianoMüller, CarstenCeballos-Laita, LuisMuñoz Pérez, IkerCeylan, BayramMustad, VikkieChen, Si-TongNagaraja, Mamta PatelChen, Yung-ShengNavarro-Flores, EmmanuelChethan, K. N.Neiva, Henrique P.Chiementin, XavierNikolaos, KoutlianosCholewa, JarosławNorberg, MichaelChtourou, HamdiPacheco, Matheus MaiaClemente-Suarez, Vincente JavierPadulo, JohnnyCoco, MarinellaPalermi, StefanoCoertjens, MarceloParpa, KoullaColl, JesúsPaternostro, FerdinandoComfort, PaulPattappa, GirishCondello, GiancarloPavlu, DagmarCzogała, WojciechPelletier, ChelseaDe Matos, DihogoPerkins, RyanDe Sá Ferreira, ArthurPessôa Filho, DaltonDemarie, SabrinaPetrigna, LucaDi Cagno, AlessandraPezarat-Correia, PedroDias, GonçaloPezzanite, Lynn M.Doherty, ColinPicerno, PietroDonahue, PaulPietrangelo, TizianaDonti, AnastasiaPimentel, GustavoDwyer, Maureen K.Pinheiro, JoãoEckerson, Joan M.Pregowska, AgnieszkaEsposito, LucaRebelo-Gonçalves, RicardoFerreira, Rogério ManuelRefoyo, IgnacioFirek, AnthonyRiebe, DeborahFouasson-Chailloux, AlbanRodacki, AndreFranzoni, FerdinandoRodríguez-Sanz, JacoboFry, AndrewRoman, MihaiGalli, FedericaRomero-Franco, NataliaGamonales, Jose M.Rosenbaum, DieterGatz, MatthiasRossi, Stefano Marco PaoloGeorge, KoumantakisRuivo Alves, AnaGhigiarelli, Jamie J.Sambri, AndreaGomes, BeatrizSánchez-Romero, Eleuterio A.Göpfert, BeatSantangelo, Kelly S.Graça, Flávia AparecidaSanti, KristiGronek, PiotrSarmento, HugoGualdi-Russo, EmanuelaSathish, MuthuGuillen-Grima, FranciscoSefton, Jo EllenHaragus, HoriaSilva, LuísHernández-Lepe, Marco AntonioSilveira, Erika AparecidaHostiuc, SorinŠimenko, JožefHsueh, Ming-FengSnih, Soham AlHuber, ReneSolarino, GiuseppeIliescu, Madalina-GabrielaSousa, AntónioIndino, CristianStanford, JohnIshida, AiStastny, PetrJaszczur-Nowicki, JarosławStavropoulos-Kalinoglou, AntoniosKarczmarska-Wódzka, AleksandraStoll, SharonKazimierczak, ArkadiuszStone, Michael H.Kellis, EleftheriosStruzik, ArturKim, HyunjoongSuchý, JiříKim, Soo-YeonSudhadevi, TaraKing, Adam C.Suguru, OhsawaKleinöder, HeinzSzabo, TamasKluess, HeidiTam, Hon LonKulesskaya, NataliaTancredi, VirginiaKumar, RavinderTarantino, DomizianoKump, DavidTavares, PaulaKwong, PatrickTee, VincentLacerda, Ana Cristina RodriguesTiefenboeck, Thomas M.Lakicevic, NemanjaTomescu, Mirela CleopatraLandram, Michael J.Toselli, StefaniaLane, AndrewVaquero-Cristobal, RaquelLatino, FrancescaVasconcelos, CarlosLavender, AndrewVerma, Vijay KumarLeirós-Rodríguez, RaquelVila-Chã, CarolinaLichtenberg, SvenVillarejo Mayor, John JairoLinek, PawełWillems, Mark Elisabeth TheodorusLipert, AnnaWomack, Christopher J.Lochbaum, MarcWurm, MarkusLolis, Evangelos D.Yadav, VirendraLópez Garcia, José ManuelYadava, Ramesh S.López Gullón, José MaríaYe, LiangLópez-de-Celis, CarlosYoshio, NakataLosa Iglesias, Marta ElenaZhang, YanjieLouder, TalinZhao, XiangliLucidi, FabioZieliński, Grzegorz


